# Colorimetric Detection of Arsenic (III) and Mercury (II) Ions in Human Serum Albumin Samples Using Cysteine-Capped Gold Nanoparticles

**DOI:** 10.3390/s26092875

**Published:** 2026-05-04

**Authors:** Sayo O. Fakayode, David K. Bwambok, Eris Arth, Ufuoma Benjamin, Rebecca Huisman, Allison Lugue, Alex Tokos, Kayley Owens, Peter Rosado Flores

**Affiliations:** 1 Department of Chemistry and Physics, College of Arts and Sciences, University of North Carolina at Pembroke, Pembroke, NC 28372, USA; 2 Department of Chemistry, Ball State University, Muncie, IN 47306, USA; 3 Department of Chemistry, Physics and Astronomy, Georgia College and State University, Milledgeville, GA 31061, USAkayley.owens@bobcats.gcsu.edu (K.O.);

**Keywords:** gold nanoparticles, sensors, human serum albumin, As (III) and Hg (II) ions, colorimetric detection, circular dichroism

## Abstract

**Highlights:**

**What are the main findings?**

- CysAuNPs are used for colorimetric detection of As (III) and Hg (II) Pin serum albumin.
- XRF enables the qualitative detection of As (III) and H Hg (II) ions in serum albumin.
- The binding of As (III) and Hg (II) ions resulted in serum albumin conformational changes.
- LOD, as low as 0.02 ppm for Hg (II), demonstrates the method’s sensitivity.
- The accurate prediction of As (III) and Hg (II) for > seven months without recalibrations.

**What are the implications of the main findings?**

- New alternative for rapid detection of As (III) and Hg (II) ions in physiologically relevant biological samples.
- This detection method allows for improved accessibility through lower costs and ease of use.

**Abstract:**

A continued interest in developing a low-cost, rapid screening method for quantifying Hg (II) and As (III) in biological samples stems from the toxic effects of human exposure to these heavy metal ions. This study reports the use of cysteine-capped gold nanoparticles (CysAuNPs) for chemical sensing, colorimetric detection, and quantification of As (III) and Hg (II) ions in human serum albumin (HSA) under physiological conditions. Zeta potential measurements indicated that the CysAuNPs have a negative surface charge, which was decreased in the presence of HSA and reversed to a positive value upon binding of As (III) and Hg (II) metal ions. Circular dichroism (CD) spectroscopy revealed changes in HSA conformation upon binding to As (III) and Hg (II) ions. X-ray fluorescence enables rapid qualitative screening for As (III) and Hg (II) ions before colorimetric quantification. The figures of merit (R^2^ ≥ 0.940) and the low detection limits (0.05 ppm for As (III) ions and 0.02 ppm for Hg (II)) in serum albumin demonstrate the high sensitivity of the method. The developed calibration curves correctly quantified the concentration of As (III) and Hg (II) ions of independently prepared test validation samples in HSA with an accuracy of ≥95% over a period of seven months without recalibrations, demonstrating the stability of CysAuNPs in solution and the robustness of the method for analysis of As (III) and Hg (II) ions in serum albumin.

## 1. Introduction

A continued increase in anthropogenic activities, as well as industrial and manufacturing processes, contributes to the burden of heavy metal ions and toxic metalloid pollution, leading to unintended environmental contamination and degradation consequences. Dietary intakes, hand-to-mouth ingestion, medical and dental procedures, and occupational and environmental exposure all contribute to significant human exposure to heavy metal poisoning [1]. A continued interest in developing a low-cost protocol for rapid screening and determination of heavy metal ions in biological samples arises from the toxic and adverse effects of human exposure to acute and chronic concentrations of these ions.

Mercury disrupts macromolecules and mechanisms that include thiol binding, enzyme inhibition, and glutathione peroxidase inhibition, which enhances drug resistance to different cancer treatments, increases the risk of developing endothelial dysfunction, leads to a reduction in neuroprotection, and increases the risk of ischemic injury, which can worsen outcomes and conditions like strokes and heart attacks [2,3]. Acute and chronic human exposure to arsenic creates a host of health challenges, such as cardiovascular and central nervous system dysfunction, gastrointestinal discomfort, liver damage, and changes to the skin and hair [3]. Infant and adolescent exposure to heavy metal ion poisoning impacts cognitive function, children’s intelligence quotients, and children’s development [4,5,6,7,8,9,10]. Exposure to mercury and metalloid arsenic is particularly concerning due to their volatility and occurrence in elemental, inorganic, and organic forms. Elemental mercury, mercury ions, and organomercurials (methylmercury and ethylmercury derivatives) are neurotoxins [11,12] with adverse effects on the central nervous system, as well as respiratory, pulmonary, gastrointestinal, dermatologic, hematopoietic, and teratogenic effects [1]. Mercury poisoning results in a host of health challenges: fatigue, insomnia, headache, personality disorders, hearing and vision impairment, low cognitive function, dysarthria, ataxia, depression, hallucination, loss of libido, paralysis, and death [11,13]. The lighter organomercurials and elemental mercury can also cross the blood–brain barrier, leading to substantial deposits in the lysosomal dense bodies of neurons [14,15,16].

Metalloid arsenic (As) occurs in various forms, including elemental, inorganic, and organic derivatives in soil and aquatic ecosystems [17]. However, chemical interconversion may occur, depending on the prevailing environmental conditions, between inorganic and organic arsenic species [1,18]. Sources of arsenic environmental contamination include polluted surface and groundwater resources, contaminated seafood, nonferrous metal smelting, energy production from fossil fuels, coal combustion, agricultural waste, arsenic in wood preservatives, and pesticide waste disposal in landfills [18,19]. Traditional heavy metal analysis techniques [20,21,22,23] suffer from high instrument and maintenance costs, operational expenses, and high-temperature requirements [24], which pose environmental and occupational hazards to laboratory technicians [25]. These challenges may severely hinder routine volatile mercury and arsenic analysis in most research or clinical laboratories. Colorimetric detection methods are low-cost, rapid, and user-friendly, and may provide an alternative for arsenic and mercury analyses in serum proteins. Bai and colleagues [26] recently reported the use of G-quadruplex/hemin for the colorimetric detection of Hg (II) in human serum albumin, demonstrating good selectivity and a low detection limit. However, the G-quadruplex of the DNA strand requires critical optimization of catalytic activity for mercury detection in serum albumin. In addition, the reported studies did not include arsenic detection. The overarching goal of this study is to use a multifaceted analytical approach, including portable X-ray fluorescence (XRF) for rapid screening, cysteine-capped gold nanoparticle (CysAuNP) sensors for the colorimetric detection and quantification of arsenic and mercury ions in serum samples, and CD spectroscopy for probing molecular interactions and conformational changes in HSA. CysAuNP sensors are ideal for chemical sensing in biological systems under near-physiological conditions due to their ease of preparation, biocompatibility, long-term stability, non-toxicity, and optical properties [27]. In addition to its non-toxicity, one of the desirable properties of cysteine as a capping agent for gold nanoparticles is its strong binding affinity and selectivity for arsenic and mercury [28,29,30,31]. The study also focused on using portable X-ray fluorescence (XRF) for the initial screening of As (III) and Hg (II) ions in HSA, which is particularly valuable at the point of care. The use of XRF for element detection is increasingly popular in environmental [32], archeological [33], forensic [34], biomedical [35], and medical diagnostic [36] studies. We further used circular dichroism (CD) spectroscopy to investigate the effects of arsenate and mercurate ion binding on HSA conformational changes. Serum albumin and blood samples are vital in biomedical and toxicological studies as biomarkers that can reliably provide information about acute exposure to toxic element ions [37]. Blood levels of Hg below 50 μg/dL (or 0.5 ppm) are considered normal; however, elevated concentrations can be observed for a couple of days after consuming seafood [38]. The elimination of Hg via the urinary tract without chelation typically does not exceed 50 μg/day; however, observable symptoms occur at mercury levels above 300 μg/L [39,40]. The study’s results demonstrated high accuracy and sensitivity for the detection of arsenic and mercury ions in serum albumin, as well as the long-term stability and applicability of CysAuNPs under near-physiological conditions.

## 2. Materials and Methods

### 2.1. Chemicals, Consumables, and Sample Preparation

Lyophilized human serum albumin (HSA) (agarose gel electrophoresis, purity > 97%), 3-(*N*-morpholino) propanesulfonic acid (MOPS, purity > 99.5%), L-cysteine, and gold tetrachloride (HAuCl_4_) were purchased from Sigma-Aldrich (St. Louis, MO, USA) and used as received. As (III) and Hg (II) element reference standards (1000 ppm %) were purchased from Fischer Scientific (Fair Lawn, NJ, USA). A lyophilized HSA stock solution and working range serum albumin solutions were prepared in MOPS buffer (30 mM, pH 7.4), as previously described elsewhere [41]. MOPS was selected for sample preparation as it prevents the denaturation of serum proteins and mitigates matrix effects in protein assays [42]. The working range for As (III) or Hg (II) ion concentrations was prepared from 1000 ppm of As (III) or Hg (II) reference standard solutions. The absorption spectra were collected using a UV–visible spectrophotometer (Shimadzu 2600i) with a temperature-controlled device.

### 2.2. Preparation of Nanoparticle Samples, Characterization, and Instrumental Analysis

A Zetasizer Nano ZS dynamic light scattering (DLS) (Malvern Instruments, Westborough, MA, USA) was used to characterize the diameter and surface charge (zeta potential) of the CysAuNPs. A Transmission Electron Microscope (TEM) imaging (JEOL, Tokyo, Japan) (accelerating voltage, 120 kV) was used to characterize the size and morphology of CysAuNPs, as reported by [41]. The UV–visible spectra were recorded using a UV-2600i UV–visible spectrophotometer (Shimadzu, Kyoto, Japan), with a temperature-controlled device accessory, scanning the 200–600 nm wavelength range (bandwidth, 2.5 nm; quartz cuvette, 1 cm path length). The sample was equilibrated for one hour to allow for the binding of As (III) or Hg (II) ions to serum albumin, before all measurements were taken at physiological conditions of 37 °C and pH 7.4. The X-ray fluorescence (XRF) sample measurement was performed by filling XRF sample cups (30.7 mm OD × 22.9 mm high, 1330-SE, Chemplex Industries Inc., Palm City, FL, USA) with an HSA sample containing known concentrations of As (III) or Hg (II) ions. The sample was covered with a Prolene 4.0 µm thin film (Complex Industries Inc., Palm City, FL, USA). The XRF measurements of As (III) and Hg (II) in serum albumin solutions were analyzed using an Olympus X-ray fluorescence analyzer (Vanta Max Series, Evident Scientific Inc., Waltham, MA, USA) via a Geochem (3-Beam) setting (50 kV three beam). Sample circular dichroism (CD) spectra were measured using a CD spectrometer (JASCO-1100 CD, Japan). The spectrometer was purged with nitrogen gas before the sample measurement and maintained under a nitrogen atmosphere during the sample measurement.

## 3. Results and Discussion

### 3.1. Characterization of the CysAuNPs, and HSA–CysAuNPs with As (III) and Hg (II) Ions

**Figure 1:**
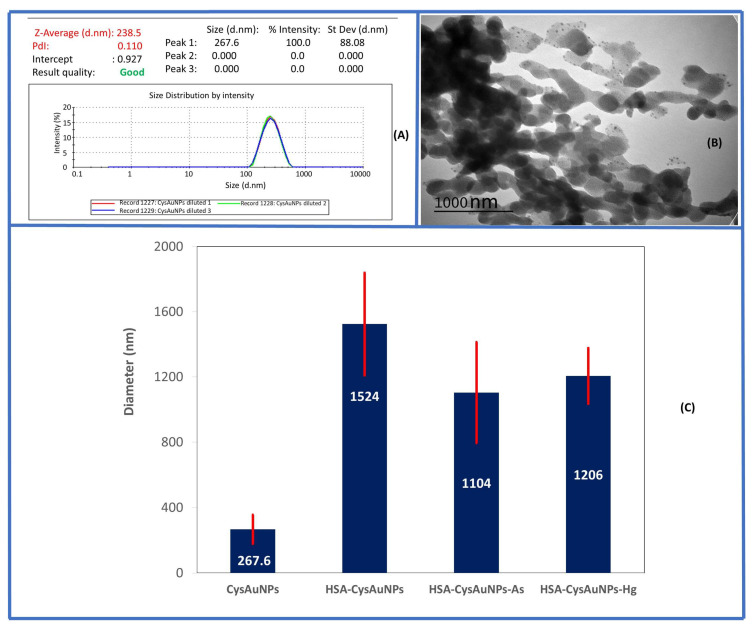
(**A**) Dynamic light scattering (DLS) measurement of CysAuNPs in water showing an average hydrodynamic diameter of 238 nm. (**B**) TEM of CysAuNPs (at 120 kV, 300 K magnification) showing spherical, nanoplate-like morphology of CysAuNPs. (**C**) Comparison of the diameter of CysAuNPs showing an increase in hydrodynamic diameter of the particles in HSA and with As (III) and Hg (II), possibly due to protein- and metal-induced aggregation. The error bars represent standard deviation from triplicate measurements (N = 3).

Dynamic light scattering (DLS) measurements of diluted CysAuNP dispersions showed a hydrodynamic diameter of 238 nm and a polydispersity index (PDI) of 0.110 (Figure 1A), suggesting more monodisperse nanoparticles. Transmission electron microscopy (TEM) of CysAuNPs revealed a spherical to nanoplate-like morphology, as shown in Figure 1B.

**Figure 2:**
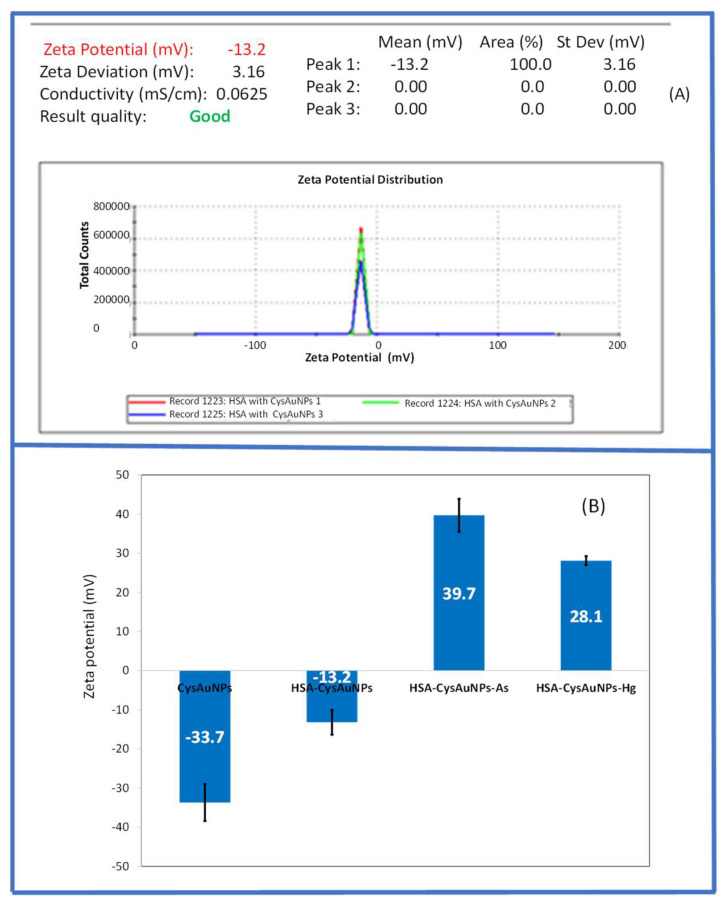
(**A**) An example of zeta potential of HSA–CysAuNPs with a Z-value of −13.2 mV. (**B**) A graph showing a comparison of zeta potentials for CysAuNPs in HSA and with As (III) and Hg (II) ions. The zeta potential measurements reveal the sign of the surface charge was reversed (from negative to positive) and the variation in charge density of HSA–CysAuNPs occurred following functionalization with As (III) and Hg (II) ions. The error bars represent standard deviation from triplicate measurements (N = 3).

The shape of CysAuNPs observed by TEM, as shown in Figure 1B, is similar to that reported in previous studies [43]. The CysAuNPs in HSA with As (III) and Hg (II) ions had a larger hydrodynamic radius of >1000 nm (Figure 1C), which can be attributed to aggregation induced by HSA proteins complexed with heavy metal ions. This result is supported by the higher PDIs for these HSA–protein complex nanoparticles (ranging from 0.248 to 0.288), compared to the PDI of 0.11 for free CysAuNPs. Surface charge is a valuable characteristic of nanoparticles that can shed light on the interaction mechanisms between CysAuNPs and HSA for the detection of As (III) and Hg (II) ions. The zeta potential measurement showed that CysAuNPs have a highly negative surface charge of −33 mV, indicating that they are stable in water (Figure 2). Interestingly, the negative charge decreases from −33 mV to −13 mV when CysAuNPs are dispersed in the HSA solution. Even more interestingly, the surface charge was reversed to a positive value upon the addition of heavy metals, reaching 39 mV for arsenic and 28 mV for mercury ions. These results suggest that the binding of HSA to CysAuNPs and the metal ions results in the cationic groups residing on the surface of the complex. In addition, the high surface charge of As (III) complex compared to that of Hg (II) may be attributed to the higher oxidation state of As (III) than that of Hg (II) ions.

### 3.2. Qualitative Detection of As (III) and Hg (II) Ions in Serum Albumin by XRF

In the initial study, we used a portable XRF instrument for rapid screening of As (III) or Hg (II) ions in serum samples, followed by colorimetric quantification of As (III) or Hg (II) ions in HSA samples. We used a commercially available lyophilized HSA in this study to alleviate the need for the isolation of serum albumin from human serum or whole blood. HSA samples at varying known concentrations (0 ppm to 50 ppm) of either As(III) or Hg(II) were subjected to XRF analysis. The portable XRF spectrometer detected As (III) and Hg (II) ions in serum samples at relatively high concentrations with reasonable accuracy, as evidenced by LODs of 16 ppm and 29 ppm for As (III) and Hg (II), respectively. The sensitivity and limit of detection of the XRF instruments varied and were instrument- and element-specific [44]. The LOD by XRF for arsenic detection in this study is comparable to the levels reported by Capobianco et al. for *Pteris vittata* [45]. The use of a portable XRF spectrometer allowed the rapid detection, on-demand screening, and diagnosis of As (III) and Hg (II) with flexibility for use in the field, at the medical provider’s office, or in a laboratory. The XRF analysis can then be followed by accurate colorimetric quantification of As (III) and Hg (II) ions in serum albumin. There is a continued interest in the use of handheld XRF as a rapid, on-site screening tool for assessing heavy metal environmental contamination [46,47,48,49], as well as in biomedical studies [50].

### 3.3. Colorimetric Detection of As (III) and Hg (II) Ions in Serum Albumin

**Figure 3:**
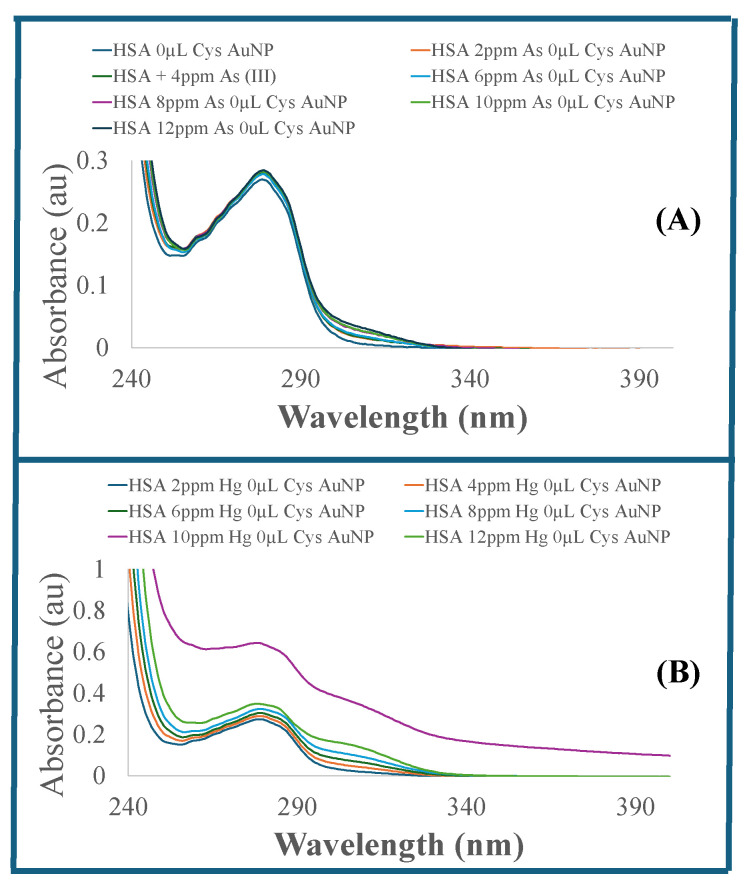
(**A**) UV–visible spectra of a solution of varying concentrations (2 ppm to 12 ppm) of As (III) ion, and (**B**) Hg (II) ion in serum albumin.

The UV–visible absorption spectra obtained for As (III)–HSA complexes of varying concentrations of As (III) ions in a fixed HSA solution are presented in Figure 3A. The significant UV–visible absorption property of HSA is attributed to the presence of tryptophan residues [51]. The solution shows a cloudy gray color change and an increase in HSA absorption intensity with increasing As (III) concentrations.

Figure 3B shows the corresponding UV–visible absorption spectra of HSA solution and Hg (II)–HSA complexes of varying Hg (II) concentration at 37 °C and pH 7.4 in MOPS buffer. The addition of increasing Hg (II) ion concentrations into HSA samples resulted in a more pronounced cloudy gray color change and higher HSA absorption, characterized by a slight hyperchromism and blue shift. The binding of As (III) or Hg (II) ions with serum albumin was facilitated by sulfur on serum albumin’s cysteine residues. Based on Pearson’s hard–soft acid–base theory [52], hard acids prefer binding to hard bases, forming ionic complexes. In contrast, soft acids preferentially bind to soft bases to give covalent complexes. As (III) and Hg (II) are soft acids that preferentially bind to the sulfur (S) available on HSA by a single free thiol group from cysteine. In addition, the interaction is covalent, and its strength depends on the metal ion’s charge-to-size ratio. Hg (II) is a softer metal ion than As, and more electropositive with a larger metal diameter; therefore, it forms a stronger covalent interaction with the S atom. Studies in the literature have reported the preferential binding of As (III) and Hg (II) ions at the free Cys34 site of HSA [53,54,55]. Arsenic is known to strongly bind to serum albumin protein [56,57]. Other studies have reported strong binding affinities and thermodynamic properties in As (III)–serum interactions [58].

**Figure 4:**
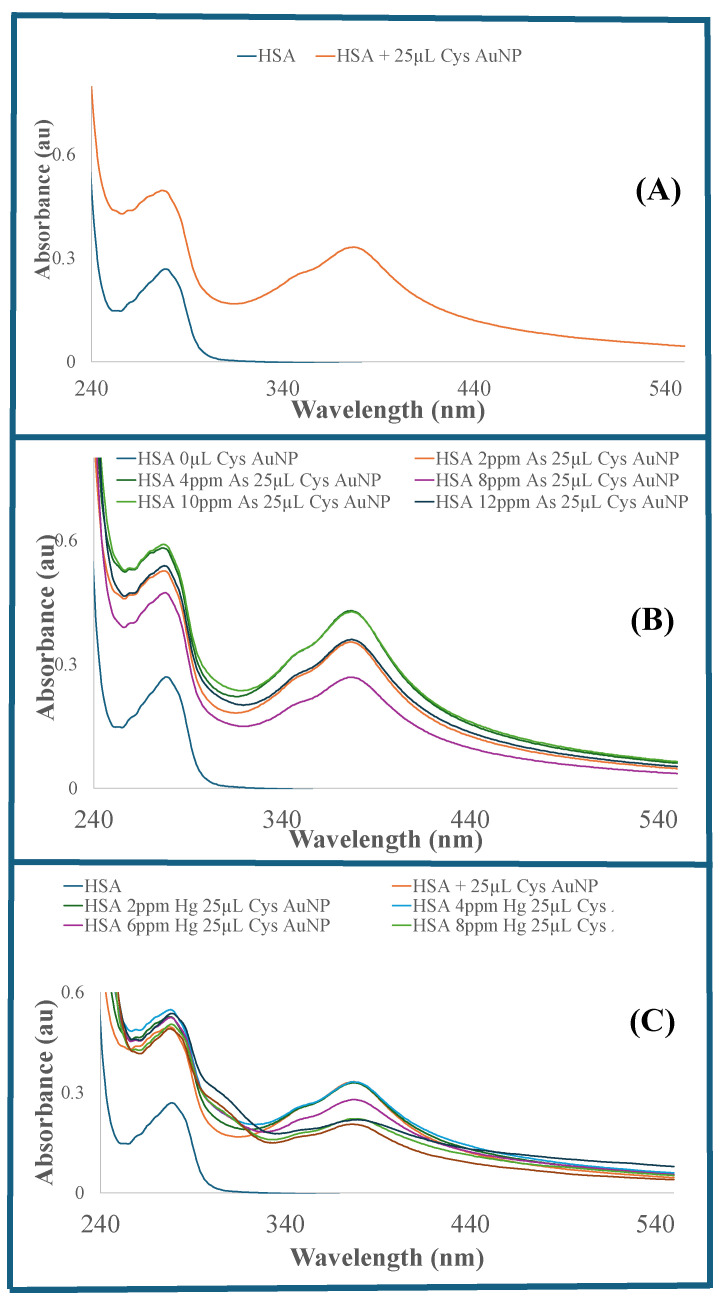
(**A**) UV–visible absorption of HSA and CysAuNPs solution. (**B**) UV–visible absorption of solutions of varying As (III) ion concentrations (2 ppm to 12 ppm) in serum albumin with the addition of 25 µL of CysAuNPs (LOD = 0.02 ppm). (**C**) UV–visible absorption of solutions of varying Hg (II) ion concentrations (2 ppm to 12 ppm) in serum albumin with the addition of 25 µL of CysAuNPs (LOD = 0.05 ppm).

The reported negative free energy, negative low entropy, and positive entropy change in the literature are indicative of the high favorability of the As (III)–serum interaction, which is reportedly governed by ionic interactions [57]. Studies in the literature have also revealed the binding of As (III) to serum proteins at the Amide I (1657 cm^−1^) and Amide II (1545 cm^−1^) bands, along with a strong As (III)–serum protein binding constant and affinity of As (III) with serum proteins [56,57,58,59]. The binding of As (III) or Hg (II) ions to serum albumin can alter the conformation and stereochemistry of HSA, ultimately leading to protein agglomeration [25]. Previously reported spectroscopic studies (UV–visible, Raman spectroscopy, and circular dichroism) have also shown conformational changes in serum upon binding to Hg (II) ions [60]. Spectroscopic and thermodynamic studies have also demonstrated a strong binding affinity of Hg (II) for two binding sites on serum albumin, leading to conformational changes in the serum protein upon Hg (II) binding [60,61]. An increase in Hg (II) concentration altered the CD spectral profile and the intensity of serum albumin. These changes are attributed to a decrease in the α-helix content of serum proteins at higher Hg (II) concentrations [60,62]. A cloudy agglomerated HSA was observed at higher Hg (II) concentrations (≥10 ppm), which could affect serum physiology and functionality, with potential adverse health implications for humans. For instance, HSA agglomeration may impair the serum’s ability to transport and distribute metabolites to their targets. Changes in the HSA tertiary structure could potentially suppress its chiral recognition property, a critical aspect of the drug, as well as the binding and efficacy of chiral metabolites. The UV–visible absorption spectra of HSA and HSA with 25 µL CysAuNPs are shown in Figure 4A. The addition of CysAuNPs to the HSA solution increased the intensity of the HSA UV–visible absorption spectra, accompanied by a blue spectral shift. This observation was consistent with prior UV–visible absorption studies of CysAuNPs–HSA [63]. Figure 4B shows the UV–visible absorption of a solution of varying As (III) ion concentrations in serum albumin with the addition of 25 µL of CysAuNPs. The corresponding UV–visible absorption of solutions across a range of Hg (II) ion concentrations (2 ppm to 12 ppm) in serum albumin with the addition of 25 µL of CysAuNPs is presented in Figure 4C. An increase in the concentration of As (III) or Hg (II) ions in the presence of CysAuNPs resulted in dramatic changes in the UV absorption of serum albumin at 240 nm and 380 nm.

### 3.4. Quantifications of As (III) and Hg (II) Ions in Serum Samples

**Figure 5:**
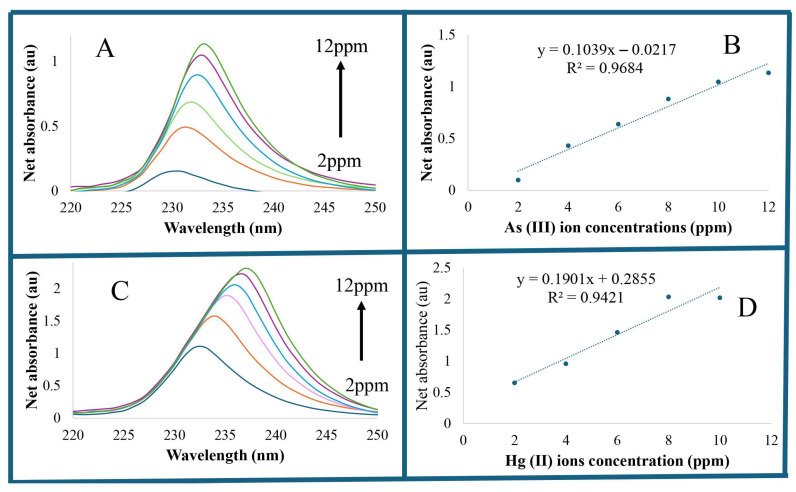
(**A**) As (III) net absorption spectra. (**B**) As (III) ion calibration curve in serum albumin. (**C**) Hg (II) ion net absorption spectra. (**D**) Hg (II) ion calibration curve in serum albumin.

The practical application of analytical methods for quantifying As (III) and Hg (II) ions in serum albumin was demonstrated using independently prepared test validation solutions, with known As (III) and Hg (II) ion concentrations prepared from a reference standard in serum albumin. Due to the high UV–visible absorption of the HSA–CysAuNPs solution, it is pertinent to determine the net absorbance (differences between the absorbance of HSA–CysAuNPs and HSA–CysAuNPs–As (III) solution for each As (III) ion concentration) of metal ions–CysAuNPs solution. Figure 5A shows the net absorbance spectra of As (III)–CysAuNP ions in serum albumin. Net absorbance values are subsequently used to construct the calibration curves. Figure 5B shows the constructed calibration curve for As (III) ions in serum albumin.

Figure 5C,D shows the net absorbance spectra of Hg (II)–CysAuNP ions in serum albumin and the resulting calibration curves. The figures of merit (R^2^ > 0.940) and low limits of detection (*3s*/*m*), where *s* is the standard deviation of the blank solution in triplicate analysis, and *m* is the slope of the regression line [64] of 0.05 ppm for As (III) and 0.02 ppm for Hg (II), demonstrate the high sensitivity of the developed method. The LOD obtained from these results is comparable to or lower than the reported LOD value of 0.026 ppm of ICP-MS analysis of arsenic in soil and sediments [65,66]. In addition, the LOD of Hg (II) detection in serum albumin is comparable to the reported LOD value of 0.0079 μg/L and LOQ values of 0.026 μg/L for ICP-MS of canned tuna fish of the Persian Gulf mercury analysis [67].

### 3.5. Method Validation Study

**Figure 6:**
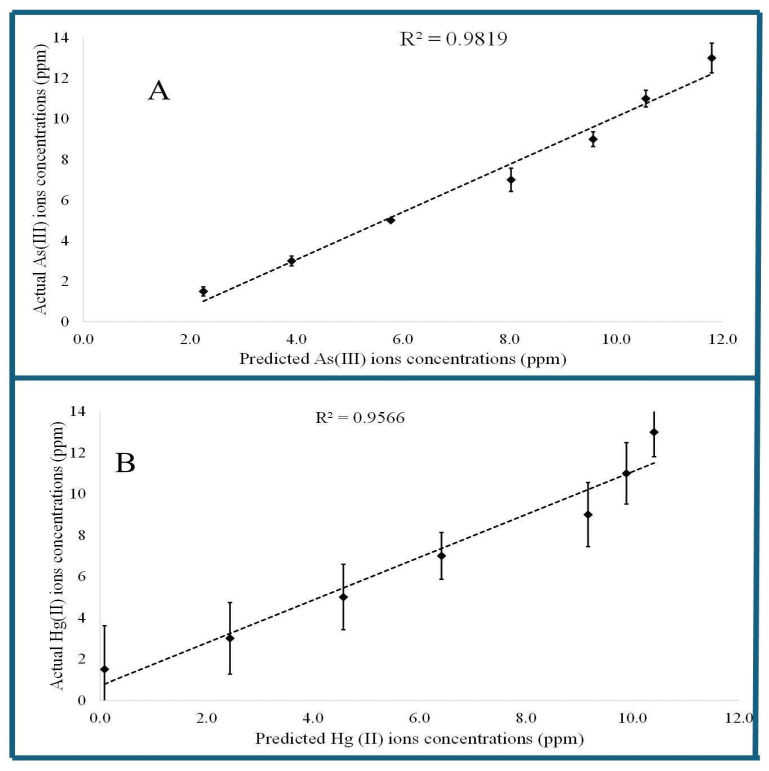
Validation study of (**A**) As (III) ion; and (**B**) Hg (II) ion in HSA–CysAuNPs samples. Error bars represent standard deviation from three independent measurements (*n* N= 3).

The constructed calibration curves were used to quantify the independently prepared test validation samples of As (III) and Hg (II) ion concentrations. Figure 6 shows the plot of actual and determined As (III) and Hg (II) ions concentration in HSA samples of the three independent trial validation samples. The accuracy of the developed CysAuNP sensors for determining As (III) and Hg (II) ion concentrations in serum albumin was evaluated by calculating the relative root mean square % relative errors (RMS%RE). The developed calibration curves accurately predicted the Hg (II) and As (III) ion concentrations of independently prepared test validation samples in serum albumin with an accuracy of greater than 95% for more than seven months without the need for recalibration, demonstrating the stability of CysAuNPs in solution and the robustness of the developed method for As (III) and Hg (II) ions analysis in serum samples.

This study’s accuracy, robustness, and low cost are impressive. However, one limitation of the method is the use of lyophilized HSA solutions in the study, rather than real human serum or whole blood. Isolation of serum albumin in whole blood introduces additional sample preparation steps that may limit the rapid application in point-of-care settings. Also, the presence of competing metal ions in whole blood may impact the analytical selectivity for the detection of As (III) and Hg (II) ions.

### 3.6. CD Study

**Figure 7:**
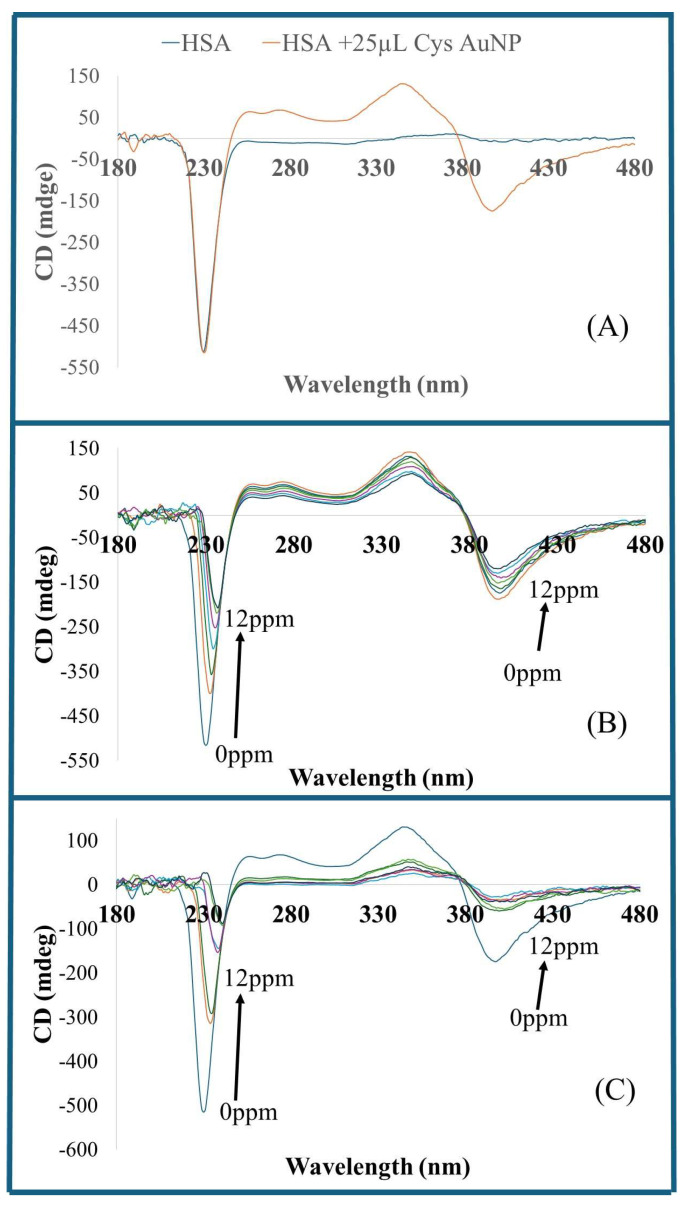
CD spectra of (**A**) HSA and HSA–CysAuNPs solution; (**B**) HSA samples containing varying concentration of As (III) ions; and (**C**) HSA samples containing varying concentration of Hg (II) ions.

We further utilized CD spectroscopy to investigate the impact of As (III) and Hg (II) ion binding on HSA stereochemistry and conformational changes. Figure 7A shows the CD spectra of HSA and HSA solution with the addition of 25 µL CysAuNPs. The interaction of CysAuNPs does not affect the HSA CD spectra at 230 nm. However, in addition to the HSA CD spectrum at 230 nm, HSA samples with CysAuNPs showed two additional CD peaks at 348 nm (positive) and 397 nm (negative). These peaks may be credited to the CysAuNPs CD spectra. Figure 7B shows the CD spectra of HSA solutions of varying As (III) concentrations and 25 µL CysAuNPs showing the HSA wavelength maxima at 230 nm, and CD spectra peaks at 348 nm (positive) and 397 nm (negative) that are attributed to CysAuNPs CD spectra absorption. Figure 7C shows the CD spectra of HSA of varying Hg (II) concentrations and 25 µL CysAuNPs showing the CysAuNPs CD spectra at wavelength maxima at 348 nm (positive) and 397 nm (negative).

While CysAuNPs have no notable impact on HSA CD spectra absorption at 230 nm, increased concentrations of As (III)–HSA and Hg (II)–HSA CD spectra absorption, as well as an increase in As (III) or Hg (II) concentrations in HSA samples, affected the HSA CD spectra at 230 nm, where the wavelength maxima were prevalent in the HSA samples. A similar change in the CD spectra of serum albumin with increasing Hg (II) concentrations have been reported, attributed to a decrease in the α-helix content of serum proteins at higher Hg (II) concentrations [56,58].

**Figure 8:**
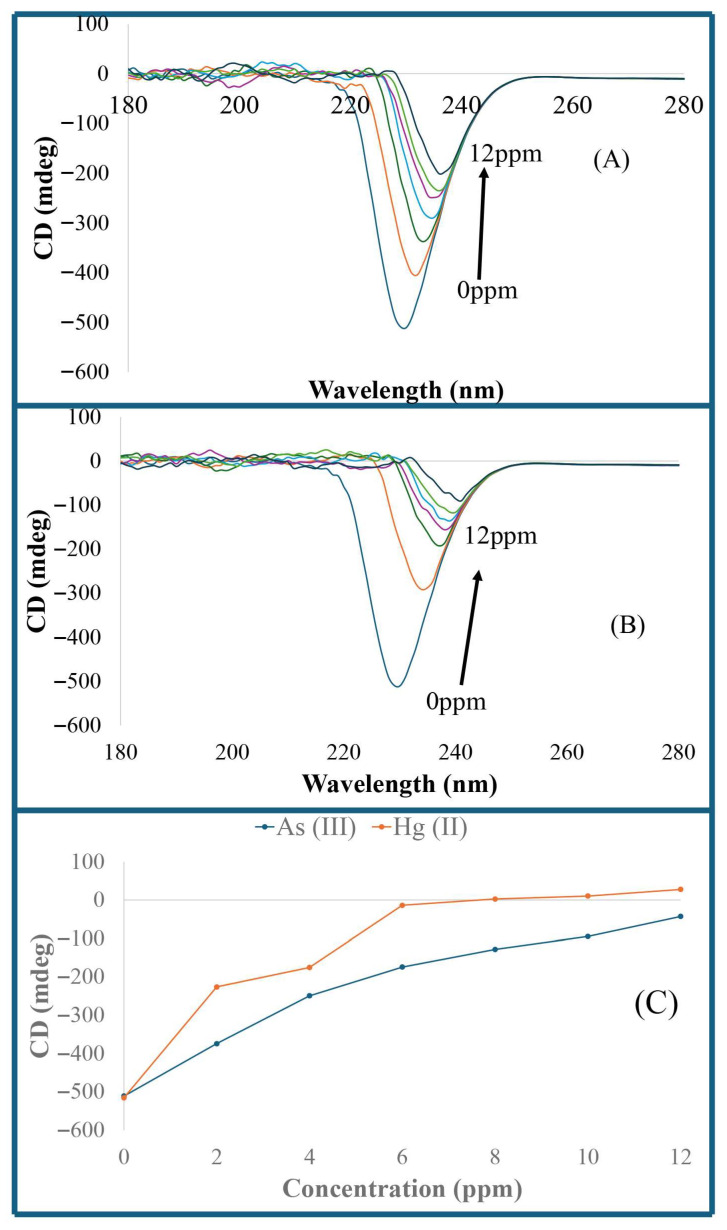
CD spectra of HSA containing varying concentrations of (**A**) As (III) ions; and (**B**) Hg (II) ions. (**C**) Overlay of the intensity of CD spectra of HSA containing varying concentrations of As (III) and Hg (II) ions at 230 nm.

Figure 8A shows the CD spectra of has, demonstrating the ellipticity of the band and orientation in circular dichroism of HSA and As (III)–HSA complexes. HSA has a CD spectrum with λ_max_ of 230 nm. The intensity of the HSA CD spectra increases notably from −503 mdeg with an increase in As (III) concentration in the HSA samples. Additionally, the HSA sample containing 12 ppm of As (III) ions showed a significant shift in the CD spectra from 230 nm to 236 nm. Figure 8B shows CD spectra of HSA containing varying Hg (II) concentrations. Again, the interaction of Hg (II) ions with HSA increased the intensity of the HSA CD spectra, accompanied by a significant spectral shift (up to 12 nm). A comparison of the intensity plot of CD spectra (at 230 nm) of solutions containing varying concentrations of As (III)–HSA and Hg (II)–HSA complexes is shown in Figure 8C. Compared to As (III), the binding of Hg (II) with HSA has a greater impact on the HSA CD spectra, which is also consistent with the result of As (III)–HSA and Hg (II)–HSA UV–visible absorption spectra.

## 4. Conclusions

CysAuNP sensors were prepared and utilized for the colorimetric detection and quantification of Hg (II) and As (III) in HSA. The addition of CysAuNPs increased the intensity of the absorption spectra of serum albumin. The binding of As (III) and Hg (II) with HSA resulted in changes in the UV–visible absorption and CD spectra of serum albumin, indicating conformational and structural changes in the protein. The availability of portable colorimetric instruments will facilitate rapid on-demand screening and detection of As (III) and Hg (III) in serum albumin in the field, at a medical provider’s office, or in a laboratory. The figures of merit are evident from the high correlation coefficients and low limit of detection, highlighting the method’s linearity and high sensitivity. CysAuNPs are stable in solutions and can be reused for the quantification of As (III) and Hg (II) ions in serum samples for over seven months without the need for recalibration, demonstrating the long-term stability of the CysAuNP sensor system and its applicability under near-physiological conditions. The sensitivity and stability of CysAuNPs in solution: the method’s robust capability of detecting as low as 0.05 ppm for As (III) ions and 0.02 ppm for Hg (II) ions, present an opportunity and alternative option for rapid detection of As (III) and Hg (II) ions in serum and biological samples at physiologically relevant conditions. Further studies include the metal ion selectivity and use of CysAuNP sensors for the multicomponent analysis of Hg (II) and As (III) ions in serum albumin using multivariate calibration approaches in biological matrices.

## Data Availability

The raw data supporting the conclusions of this article will be made available by the authors on request.
